# Prevalence of Self-Medication Among Patients With Common Dermatological Diseases in Saudi Arabia

**DOI:** 10.7759/cureus.62325

**Published:** 2024-06-13

**Authors:** Abdulaziz A. Aljuaid, Mohammad Basendwh, Hamid S Alhamid, Esraa A Shaheen, Rakan S. Alajmi, Amal H Abualola

**Affiliations:** 1 College of Medicine, King Saud Bin Abdulaziz University for Health Sciences, Jeddah, SAU; 2 Department of Dermatology, King Fahad Armed Forces Hospital, Jeddah, SAU; 3 Department of Dermatology, Ministry of National Guard, National Guard Hospital, Riyadh, SAU

**Keywords:** skin disorders, dermatological diseases, prevalence, dermatology, self-medication

## Abstract

Introduction

Self-medication is defined as the use of a medication or product to treat, alleviate, or prevent a condition or symptom of an illness or to promote health. Self-management is increasingly used for dermatological diseases, especially chronic inflammatory skin diseases. Hence, it is crucial to be aware of its prevalence and the leading factors of its usage. Therefore, this study aims to estimate the prevalence of self-treatment for dermatological diseases and to determine possible factors associated with its practice.

Methodology

This is a cross-sectional online-based questionnaire study that was conducted in King Fahad Armed Forces Hospital in Jeddah, Saudi Arabia. In this study, we included all first-visit and follow-up patients diagnosed with vitiligo, eczema, alopecia areata, and psoriasis. Data analysis was conducted using JMP Statistical Discovery Software, version 15 (JMP Statistical Discovery LLC, Cary, NC).

Results

Of the 203 patients who participated in this study, 91 (44.8%) had vitiligo. Moreover, topical corticosteroids were the most commonly used medications in self-treatment and included mometasone and hydrocortisone, which were used by 143 (41.3%) and 50 (14.5%) patients, respectively. Thirty patients (16.2%) improved with self-treatment, whereas 52 (28.1%) had no improvement. In addition, the most common reason for self-treatment was having mild symptoms of the disease in 97 patients (30.1%).

Conclusion

This study revealed that the prevalence of self-treatment among various dermatological diseases was 53.2%. Topical corticosteroids were the most commonly used conventional medicines, whereas the most common alternative medications used were honey and henna. We found that the most common reason for self-treatment was mildness of the dermatological disease. Family and friends were the most common sources of information.

## Introduction

Dermatological disorders account for a significant portion of all health issues. Approximately, one out of three patients presenting to general practitioners struggle with skin disorders [1]. Most dermatological diseases are chronic and cause a significant decrease in patient-related quality of life [2]. However, some patients perceive their skin problems to be self-limited and senseless to seek medical attention, so they start self-management before seeking medical advice and do not keep an eye on possible adverse events. These adverse events include the development of bacterial resistance or a serious skin disease that is masked by minor symptoms [3].

Self-medication is defined as the use of a medication or product to treat, alleviate, or prevent a condition or symptom of an illness, and to promote health. It involves a range of practices including obtaining medications without a prescription, resubmitting old prescriptions to purchase medications, sharing drugs with family and friends, using leftover medications from storage, failing to adhere to health practitioner prescriptions either by taking inappropriate doses or refusing to take the medicine, or using natural alternatives or home remedies without an expert opinion [4]. Self-management practices are increasingly utilized for a variety of health conditions including dermatological diseases, especially chronic inflammatory skin diseases like atopic dermatitis, alopecia areata, psoriasis, and vitiligo [5].

Globally, up to 45.0% of individuals have used self-management at some point in their lives. The prevalence of self-management is considered to be high not only in the general population but also among medical students who have a prevalence as high as 90%. Medical students have an increased awareness of illnesses and drug usage, which is thought to be a contributing factor [2]. Moreover, according to a systematic review of six cross-sectional studies from multiple countries, the most common types of medications used in self-medication for various skin diseases were topical corticosteroids, antibiotics, antifungals, and moisturizing products [3]. Locally in Saudi Arabia, a study estimated that 808 out of 1901 patients with skin diseases used complementary and alternative medicine (CAM). The study also reported that honey and Zamzam water were the most common therapies used for eczema, psoriasis, vitiligo, and alopecia [4].

Not surprisingly, these huge numbers rise as the factors that facilitate the use of self-medication increase. These factors can be related to the disease itself and include the long course and chronicity of the illness [6]. For example, patients with psoriasis tend to self-medicate themselves repeatedly because of their physical appearance, the fluctuating nature of the disease, and unpleasant physical symptoms like itchiness, pain, and discomfort [7]. Other factors can be related to low socioeconomic status, social media influencers, advertisements, and recommendations of salespeople. For instance, drug advertisements and easy accessibility to skin products in pharmacies encourage the practice of self-medication [3,8]. Moreover, the COVID-19 pandemic increased the fear of contracting an infection from healthcare facilities, and self-medication was often preferred during the pandemic. People commonly are self-medicated based on medications administered for an ailment in the past, on the advice of friends and family, or purchasing over-the-counter items from the pharmacy [8].

Because self-medication is a prevalent practice that is considered an essential aspect of WHO’s self-care recommendations, it must be evaluated [3]. Over the past few years, the use of self-medication has tripled. This remarkable and quick rise in the incidence of self-management has drawn the attention of researchers not only to study its impact but also to understand and analyze the factors that led to this increase [9,10]. Therefore, it is important to expand the understanding of its prevalence and factors, but unfortunately, local epidemiological data about the use of self-management among patients with skin diseases is lacking [5].

For these reasons, this study aims to estimate the prevalence of self-management usage among dermatological diseases and to determine possible factors associated with its usage. 

## Materials and methods

Study design and setting

This is a cross-sectional online-based questionnaire study that was conducted at King Fahad Armed Forces Hospital in Jeddah, Saudi Arabia, over four months from the beginning of June until the end of September 2022. 

Participants

Eligible participants for the study included all males and females who were first-visit and follow-up patients with the following diagnoses: vitiligo, eczema, alopecia areata, and psoriasis. Participants were required to read and accurately complete a self-reported questionnaire. We excluded individuals who declined participation or who did not fully complete the questionnaire. 

Data collection

Data were collected using an online-based, multiple-choice, self-administered questionnaire that was disseminated to eligible patients attending dermatology clinics.

Variables

The questionnaire is composed of four sections. The first section includes the sociodemographic information of the patients (gender, age, income, and educational level). The second section determines the patients’ condition and the type of visit and ascertains whether the self-treatment occurred with or without consulting a doctor and before or after seeing a doctor. The third section focuses on the type of medications used (topical steroids, topical calcineurin inhibitors, and topical antibiotics). The fourth section explores the use of different cultural or herbal supplements (henna, honey, myrrh, black seeds, Rashad seeds, fenugreek, tar, and Zam Zam water). The fifth section focuses on the sources of information behind the patients’ self-medication. All patients consented before filling out the survey, and all information was kept in complete confidentiality in accordance with the ethical approval received from the IRB for our study.

Data sources and measurement

The questionnaire used in this study was designed based on similar studies and surveys to gain insight into patients' motivations for self-medications. The questionnaire was first drafted in English and then translated into Arabic to ensure comprehensibility among the local population. To confirm the accuracy and integrity of the translation, a back-translation process was performed.

The validity, feasibility, and basic reliability of the questionnaire have been evaluated by conducting a pilot study. The questionnaire was completed by 10 patients with the included dermatological diseases. The pilot study was tested on the same patients after two weeks and showed a reliability correlation coefficient of ≥0.95, suggesting its suitability for the study. Moreover, it was revised by dermatology consultants and research experts.

Sample size 

For this study, the sample size was computed using the Raosoft sample size calculator and was considered to have a 5% margin of error and a 95% confidence level. The sample size of 203 patients was attained using a non-probability sampling method.

Ethical approval 

The study proposal was approved by the King Fahd Armed Forces Hospital-Jeddah Research and Ethics Committee (Reference Ethical Number: REC 505).

Statistical analysis

All data were collected and entered into Microsoft Excel 2018 software. Data analysis was conducted using JMP Statistical Discovery Software, version 15 (JMP Statistical Discovery LLC, Cary, NC). Categorical variables were presented in frequencies and percentages. For the quantitative variables, descriptive statistics were used and shown as frequencies and percentages. Furthermore, the association between self-medication and selected variables was evaluated using contingency tables and a chi-square test for independence. A p-value less than 0.05 was considered to be statistically significant.

## Results

**Figure 1:**
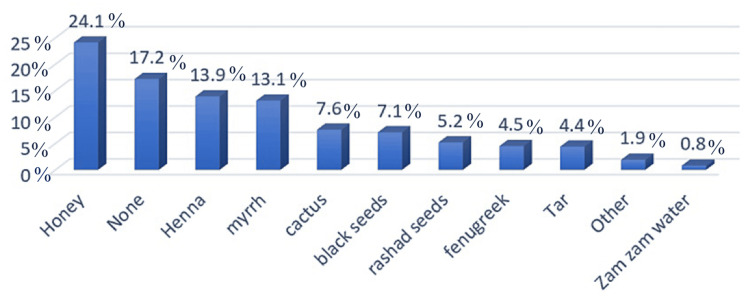
Types of cultural or herbal supplements used for self-treatment by the patients

The characteristics of the 203 patients who participated in this study are presented in Table 1. The most common skin disorder among our study participants was vitiligo, with 91 (44.8%) as demonstrated in Table 2. Patients on their first visit to the doctor numbered 47 (23.2%), and the patients who self-treated themselves without a doctor’s consultation numbered 108 (53.2%). Among the medical treatments, topical corticosteroids were the most commonly used medication and included mometasone furoate 0.1 w/w and hydrocortisone acetate, which were used by 143 (41.3%) and 50 (14.5%) patients, respectively, as shown in Table 3. Among the alternative medicines, Figure 1 shows honey and henna (derived from the *Lawsonia inermis* shrub) were the most used at 24.1 % and 13.9%, respectively. Mild symptoms of the disease 97 (30.1%), cultural and socioeconomic issues 72 (22.3%), and unwillingness to take the disease seriously 40 (12.4%) were the most frequent reasons that patients chose self-medication. Among the patients’ sources of information for self-medication, the most common were family or friends at 28.9%, prior prescriptions at 19.4%, and leftover drugs at 18.8%. Table 4 shows the basic characteristics of the participants who practiced self-treatments. Gender, age, financial status, and education level were not significant, with p-values of more than 0.05.

**Table 1:** Sociodemographic features of participants

Variable	N = 203 (%)
Gender	
Male	92 (45.3%)
Female	111 (54.7%)
Age	
<18	39 (19.2%)
18-24	39 (19.2%)
25-34	59 (29.1%)
35-45	31 (15.3%)
>45	35 (17.2%)
Financial status	
Less than 5000 Saudi Riyals	73 (35.9%)
5000-10,000 Saudi Riyals	70 (34.5%)
10,000-20,000 Saudi Riyals	47 (23.2%)
More than 20,000 Saudi Riyals	13 (6.4%)
Educational level	
Illiterate	5 (2.5%)
Elementary	7 (3.4%)
Intermediate	14 (6.9%)
Secondary	83 (40.1%)
Bachelor's	85 (41.9%)
Master's	7 (3.4%)
PhD	2 (0.9%)

**Table 2:** Diseases and self-treatment

Variable	N = 203 (%)
Diagnosed diseases	
Vitiligo	91 (44.8%)
Eczema	35 (17.2%)
Psoriasis	28 (13.8%)
Alopecia areata	35 (17.2%)
Combinations of diseases	14 (6.9%)
First visit to a doctor	
Yes	47 (23.2%)
No	156 (76.8%)
Self-treatment without consulting a doctor	
Yes	108 (53.2%)
No	95 (46.8%)
Self-treatment use (n = 102)	
Before seeing a doctor	77 (75.5%)
After seeing a doctor	25 (24.5%)

**Table 3:** Medication type and complementary medicine ^1^Multiple responses n = 346. ^2^Multiple responses n = 322.

Variable	N = 203 (%)
Use of topical steroids (n = 346)^1 ^	
Mometasone furoate	143 (41.3%)
Hydrocortisone acetate	50 (14.5%)
Betamethasone diproprionate	46 (13.3%)
Clobetasol	40 (11.5%)
None	67 (19.4%)
Use of topical calcineurin inhibitors	
Tacrolimus monohydrate (Protopic)	10 (23.3%)
Tacrolimus (Viotopic)	20 (46.5%)
Pimecrolimus (Elidel)	9 (20.9%)
Combinations	4 (9.3%)
Use of emollients	
Yes	171 (84.2%)
No	32 (15.8%)
Use of topical antibiotics	
Fucidin (fusidic acid)	97 (93.3%)
Fusiderm (fusidic acid+betamethasone valerate)	7 (6.7%)
Use of antihistamine	
Yes	102 (50.2%)
No	101 (49.8%)
Description of self-medication experience	
Significant improvement	30 (16.2%)
Moderate improvement	57 (30.8%)
Mild improvement	46 (24.8%)
No improvement	52 (28.1%)
Side effects	
Yes	46 (24.3%)
No	143 (75.7%)
Reasons for self-treatment^2^	
Mild symptoms of the disease	97 (30.1%)
Doctor’s treatment ineffective	26 (8.1%)
Availability of medications	12 (3.7%)
Inability to see a doctor	29 (9.0%)
No time to see the doctor	15 (4.7%)
Perceived benignity of the condition	40 (12.4%)
Previous prescription of the drug	28 (8.7%)
Transportation issues	5 (1.2%)
Cultural and socioeconomic issues	72 (22.3%)

**Table 4:** Self-treatment by basic characteristics of participants

Variable	Yes N (%)	No N (%)	p-Value
Gender			
Male	45 (48.9%)	47 (51.1%)	0.264
Female	63 (56.8%)	48 (43.2%)	
Age			
<18	21 (53.9%)	18 (46.1%)	0.742
18-24	17 (43.6%)	22 (56.4%)	
25-34	34 (57.6%)	25 (42.4%)	
35-45	17 (54.8%)	14 (45.2%)	
>45	19 (54.3%)	16 (45.7%)	
Financial status			
Less than 5000 Saudi Riyals	38 (52.1%)	35 (47.9%)	0.820
5000-10,000 Saudi Riyals	39 (55.7%)	31 (44.3%)	
10,000-20,000 Saudi Riyals	23 (48.9%)	24 (51.1%)	
More than 20,000 Saudi Riyals	8 (61.5%)	5 (38.5%)	
Educational level			
Illiterate	3 (60.0%)	2 (40.0%)	0.686
Elementary	2 (28.6%)	5 (71.4%)	
Intermediate	7 (50.0%)	7 (50.0%)	
Secondary	44 (53.0%)	39 (47.0%)	
Bachelor's	45 (52.9%)	40 (47.1%)	
Master's	5 (71.4%)	2 (28.6%)	
PhD	2 (100%)	0 (0%)	

## Discussion

​Even though self-treatment is an undeniable part of WHO’s self-care recommendations, it is associated with undesirable health-related consequences. For example, side effects, polypharmacy, drug-drug interactions, wasting of resources, and pathogen resistance are all common unwanted results of self-treatment [11]. Ranging from 6% to 90.09%, self-medication prevalence was notably variable in several study settings across the world [2,3]. In this paper, we measured the prevalence of self-medication among dermatological patients at 53.2%. For such a high prevalence, this study aims to shed light on self-treatment among dermatological patients. Moreover, this paper assesses the prevalence of self-treatment, the types of medications used, sources of information, and reasons to self-treat dermatological diseases.

One study revealed that topical antifungal drugs were the most common medications used by dermatological patients who practiced self-medication [2]. Another study found that topical corticosteroids were the most common medication [3]. This was mirrored in a study conducted in the same area showing allopathic medications were most commonly used by patients practicing self-medication [12]. In the current study’s setting, we found that topical corticosteroids were the most common type of medication used in self-managing dermatological diseases.

CAM can be defined as a collection of different medical systems, practices, and products that are not part of conventional medicine [13]. Alternative medical systems, mind-body interventions, energy therapies, manipulative and body-based methods, and biology-based treatments are considered the five major categories of CAM [13]. A study found that vitamins followed by prayer were the most common modalities in self-treating dermatological diseases among people using CAM [5]. Furthermore, another study revealed that honey followed by yogurt was the most frequent type of CAM used [9]. In our study, however, we concluded that honey and henna were the most common modalities of CAM among dermatological patients.

The duration of the disease, mildness of the disease, lack of health insurance, advice from family members, impact of social media, availability of alternative medications, and many other factors were important in assessing the reasons for self-medication. In this paper, we found that mild symptoms of the disease, cultural and socioeconomic issues, and failure to take the disease seriously were the most common reasons why patients sought self-medication. One study found that the mildness of the disease was the most common reason for self-treating dermatological diseases [2]. Moreover, this result was mirrored in a study that stated that mildness of the disease was the most common reason to self-treat acne vulgaris [12]. However, recommendations from family members and friends were the most common reason to self-treat in another study [6].

Family and friends followed by prior prescriptions were the most common sources of information in this study. A variety of sources were noted in different study settings, some supported the findings of the current study [14-16], others stated that textbooks were the most common source [17,18], while another found that medical staff and seniors were the most common sources [2], and a few articles found that knowledge and experience derived from prior prescriptions were the most common source of information regarding self-medication [19-21]. 

In Saudi Arabia, the internet or media was the most common source of information in different study settings [12,22,23].

Gender roles were a crucial part of assessing self-treatment, in that women practiced self-care more frequently than men [24]. This can be explained by the females’ familiarity with dermatological products, at least here locally. One study found that female patients were practicing self-management significantly more often than male patients [12]. The previous study findings also were consistent with other studies [19,25].

Limitations 

In contrast to the aforementioned studies, this paper did not find any significant differences between female and male patients regarding the self-management of their dermatological diseases. In addition, this is a survey-based study, so one of our limitations is that participants might have recall bias.

## Conclusions

This paper assesses self-treatment among dermatological patients and identifies the types of medications used, sources of information, and reasons for self-treating dermatological pathologies. Self-treatment was recognized to be a common practice among dermatological patients. In this study’s setting, we found that 53.2% of dermatological patients practiced self-treatment with no significant difference between male and female dermatological patients. Topical corticosteroids, including mometasone and hydrocortisone, were the most common type of medication used for self-treatment. Conversely, honey and henna were the most common alternative medications used. Moreover, the mildness of the dermatological disease was stated to be the most common reason for self-treatment, followed by cultural and socioeconomic issues and the unwillingness to take the disease seriously. Furthermore, family and friends followed by prior prescriptions were the most common sources of information. Even though it is unrealistic to completely stop this self-treatment practice, it is critically important to refrain from overlooking it or ignoring its serious drawbacks like polypharmacy, drug-drug interactions, pathogen resistance, and the waste of medical resources. Health campaigns to the public, direct doctor-to-patient education, and social media resources are all useful tools to effectively achieve a safe practice environment around the patient and the treating physician.
